# The Impact of Illicit Drug Use on Spontaneous Hepatitis C Clearance: Experience from a Large Cohort Population Study

**DOI:** 10.1371/journal.pone.0023830

**Published:** 2011-08-24

**Authors:** Hossein Poustchi, Saeed Esmaili, Ashraf Mohamadkhani, Aghbibi Nikmahzar, Akram Pourshams, Sadaf G. Sepanlou, Shahin Merat, Reza Malekzadeh

**Affiliations:** Digestive Disease Research Center, Shariati Hospital, Tehran University of Medical Sciences, Tehran, Tehran, Iran; University of Leuven, Rega Institute, Belgium

## Abstract

**Background and Aims:**

Acute hepatitis C infection usually ends in chronic infection, while in a minority of patients it is spontaneously cleared. The current population-based study is performed on a large cohort in Golestan province of Iran to examine the demographic correlates of Spontaneous Hepatitis C Clearance.

**Methods:**

Serum samples used in this study had been stored in biorepository of Golestan Cohort Study. These samples were evaluated for anti hepatitis C Virus by third generation Enzyme-linked immunosorbent assay (ELISA). Subjects who tested positive were then invited and tested by Recombinant Immunoblot Assay (RIBA) and Ribonucleic Acid Polymerase Chain Reaction test (PCR). If tested positive for RIBA, subjects were recalled and the two tests were re-done after 6 months. Those subjects who again tested positive for RIBA but negative for PCR were marked as cases of spontaneous clearance.

**Results:**

49,338 serum samples were evaluated. The prevalence of Chronic Hepatitis C Virus (CHCV) infection based on PCR results was 0.31%. Among those who had acquired hepatitis C, the rate of SC was 38%. In multivariate analysis, illicit drug use both Injecting Use (OR = 3.271, 95% CI: 1.784–6.000, p-value<0.001) and Non-Injecting Use (OR = 1.901, 95% CI: 1.068–3.386, p-value = 0.029) were significant correlates of CHCV infection versus SC.

**Conclusions:**

Illicit drug use whether intravenous or non-intravenous is the only significant correlate of CHCV, for which several underlying mechanisms can be postulated including repeated contacts with hepatitis C antigen.

## Introduction

Hepatitis C virus (HCV) infection is an important global health problem with estimates of 170 million HCV-infected individuals worldwide [1], [2]. Prevalence of hepatitis C all around the world ranges from 0.02% to18% [3]. In Iran, several studies have been performed on seroprevalence of HCV. Based on a comprehensive systematic review by Alavian et al. [4], the prevalence of HCV infection among the general population in 6 out of 30 provinces of Iran is 0.16%. Also the estimated seroprevalence of HCV in a more recent population-based study by Merat et al. [5] is around 0.5%, which is concordant with previous studies on blood donors (0.08%–1.3%) [6], [7].

In majority of patients exposed to HCV, the disease becomes chronic, but in a proportion of cases ranging from 14% to 45% the virus is spontaneously cleared from the body and the Ribonucleic Acid Polymerase Chain Reaction test (PCR) test becomes negative [8]–[15].

Several factors have been proposed as correlates of HCV persistence, including viral and demographic factors, and also route of disease transmission. [8]–[11], [14]–[19].

Among demographic factors, male gender [19]–[23], older age [14], [23], and ethnic background [12], [21], [24] are suggested as correlates for HCV persistence. Host genetic variation is also assumed to explain the heterogeneity in HCV persistence across individuals because such differences occur even if individuals are exposed to the same HCV strain [18], [25], [26].

Certain high risk behaviors also increase the risk of HCV persistence, namely illicit drug use (both Injecting and non-Injecting) [21], [27], alcohol consumption [12], [14], and unprotected sexual behaviors that lead to co-infection with Human Immunodeficiency Virus (HIV) and Hepatitis B Virus (HBV) [14], [28]–[30]. Still, there are major controversies on the role of these risk factors in various studies.

Along with effective prevention of HBV by universal vaccination in developing countries including Iran, HCV is becoming a more important public health concern replacing HBV [31]–[33]. There are quite a few studies that report the high rate of HCV co-infection with HBV, HIV, and venereal diseases such as syphilis in Iran [34], [35] especially among prisoners [36], which needs immediate attention. Appropriate policy-making in this regard necessitates accurate estimation of HCV burden. Distinguishing patients who spontaneously clear HCV from those who develop progressive liver disease and their risk factors is crucial for decision making.

In the current study, we have determined the rate of HCV spontaneous clearance (SC) and its demographic and host correlates in a large prospective population-based study in Iran.

## Results

All subjects whose serum had tested positive for third generation Enzyme-linked immunosorbent assay (ELISA) were recruited for baseline examination and were recalled after 6 months and no subject was missed in the prospective phase of the study. Three hundred and eighty one (381) subjects tested positive for ELISA, among them, 247 subjects were also positive for Recombinant Immunoblot Assay (RIBA), therefore 134 subjects were labeled as false positive. Chronic HCV infection (CHCV) was confirmed in 152 subjects by PCR and the 95 cases with negative PCR was labeled as SC. During repeat serological testing after 6 months all CHCV subjects remained positive and in none of SC cases did the result of the test change. The HCV seroprevalence in Golestan cohort was 0.5% based on the result of RIBA, and 0.31% based on the result of PCR and 38.5% of infected subjects had spontaneously cleared the infection.

There was no significant difference in the mean age between the 4 groups (CHCV, SC, false positives, and HCV negatives). The frequency of males was significantly higher among subjects with CHCV infection (73.7%) and cases of SC (52.6%) than false positives (47.0%) and HCV negatives (40.0%) (p<0.001).

The correlates of HCV acquisition in univariate and multivariate logistic regression were sought. The ultimate correlates in multivariate analysis were: history of blood transfusion, history of imprisonment, family history of hepatitis, cigarette smoking, Non-Injecting Drug Use (Non-IDU), and Injecting Drug Use (IDU) (table 1).

**Table 1:** Correlates of HCV acquisition in multivariate analysis.

Factor		OR	95% CI	P-Value
History of blood transfusion	No	1		
	Yes	2.65	1.27–5.55	0.010
History of imprisonment	No	1		
	Yes	2.01	1.02–3.96	0.043
Family history of hepatitis	No	1		
	Yes	3.65	1.27–10.43	0.016
Cigarette smoking	No	1		
	Yes	2.33	1.25–4.36	0.008
Non-IDU	No	1		
	Yes	2.09	1.16–3.78	0.015
IDU	No	1		
	Yes	6.92	4.00–11.97	<0.001

Abbreviations: Non-IDU: Non Injecting Drug Use; IDU: Injecting Drug Use.

The correlates of HCV persistence versus SC were sought. Results of the univariate logistic regression are demonstrated in table 2. Among significant correlates, body piercing appears to be inversely associated with HCV persistence.

**Table 2:** Correlates of HCV persistence versus spontaneous clearance in univariate analysis.

Factor		OR	95% CI	P-value
Gender	Female	1		
	Male	2.52	1.47–4.33	<0.001
Age for each 10 years		0.81	0.61–1.08	0.154
Marital status	single	1		
	married	3.33	2.12–3.57	0.002
Ethnicity	Non-Turkman	1		
	Turkman	0.68	0.39–1.19	0.184
History of blood transfusion	No	1		
	Yes	4.18	1.81–9.67	<0.001
History of hospitalization	No	1		
	Yes	1.23	0.74–2.05	0.432
History of surgery	No	1		
	Yes	0.88	0.52–1.49	0.630
History of dentistry	No	1		
	Yes	1.57	0.84–2.92	0.158
History of accident	No	1		
	Yes	1.67	0.84–3.30	0.143
War injury	No	1		
	Yes	2.49	0.80–7.74	0.115
History of imprisonment	No	1		
	Yes	3.70	1.82–7.14	<0.001
History of tattoing	No	1		
	Yes	1.70	0.44–6.59	0.440
History of traditional phlebotomy	No	1		
	Yes	1.87	0.71–4.92	0.206
History of body piercing	No	1		
	Yes	0.46	0.27–0.78	0.004
Family history of hepatitis	No	1		
	Yes	2.075	1.10–3.91	0.024
Cigarette smoking	No	1		
	Yes	2.048	1.19–3.52	0.009
Traditional tobacco smoking	No	1		
	Yes	2.137	1.09–4.18	0.027
Non-IDU	No	1		
	Yes	2.418	1.42–4.13	0.001
Alcohol consumption	No	1		
	Yes	3.061	1.21–7.74	0.018
Body Mass Index		1.00	0.95–1.05	0.997
IDU	No	1		
	Yes	14.26	7.80–26.07	<0.001

The test was performed on all 247 patients who had a true positive ELISA test (cases of chronic HCV infection plus cases of spontaneous clearance).

Abbreviations: Non-IDU: Non Injecting Drug Use; IDU: Injecting Drug Use.

In the next step, correlates of HCV persistence versus SC were sought using multiple logistic regression. The only variables that remained in the model were IDU (OR = 3.271, 95% CI: 1.784–6.000, p-value<0.001) and Non-IDU (OR = 1.901, 95% CI: 1.068–3.386, p-value = 0.029). There was no interaction between correlates and confounders.

## Discussion

To the best of our knowledge, the current study is the first that has been conducted in such a large scale on HCV spontaneous clearance in Iran. In this study we were able to demonstrate that 38.5% of HCV infected subject might clear HCV infection spontaneously which is in agreement with many previous studies [8]–[10], [12]–[14], [37]. Also we found that both IDU and non-IDU are the main correlates of CHVC that significantly decrease the chance of SC among those patients who have acquired HCV infection.

Over 50% of the total estimated 170 million HCV cases in the world occur among illicit drug users and over 75% of incident infections occur in this population [3]. It is known that, illicit drug use is the major correlate of HCV persistence [21], [23], [37]. Several hypotheses have been suggested to explain the underlying mechanisms of this finding which mainly include: inefficient immunological response [38] which is due to HIV in co-infected subjects [12], [14], [39] other co-infection or co-morbidities [40], [41], and finally repeated -infection [27].

As for IDU, frequent monitoring of HCV infection status among those who clear a primary infection demonstrated that re-infection of HCV following control of a primary infection is common [42], with detection of one or more subsequent infections in almost 50% of cleared subjects [43]. In a study by Aitken and colleagues, re-infection was documented in 46% of previously cleared IDUs [44]. This data suggests that prior clearance of HCV infection may not provide immunity against re-infection. However it is of interest to know that the rate of self limited infection in subjects who previously cleared the virus is higher than subjects who do not clear the virus after initial infection [43], [45]. Furthermore, maximum viral load and also duration of viremia in re-infected cases are lower compared to the primary infection [43].

Our results are in discordance with findings of Lewis-Ximenez et al. [46], who found no relationship between illicit drug use or age and rates of SC. The only correlate of CHCV infection reported by them was low HCV antibody values [46]. The insignificant results in this study may have been due to the small sample size. Similarly, Santantonio et al. [47] found no demographic factors as correlates of HCV persistence. The possible explanation is that IDUs in Italy may be less likely to reuse syringes for drug injection.

Our results on the role of Non-IDU in HCV persistence are in accord with previous studies [21], [27], [48], but there are no agreed upon mechanism for this finding. Some studies imply that nasal inhalation or oral exposure may be plausible routes of transmission probably through shared intranasal canola contaminated with blood [48].

In our study, the seroprevalence of HCV based on RIBA is 0.5%, which is similar to that reported by Merat et al. [5]; however, the seroprevalence of HCV in our results based on PCR is 0.31% which is again similar to that reported by Alavian et al. [4]. The risk factors that correlate with HCV acquisition in our study (table 1) are also similar to many previous reports.

In the current study, the rate of SC is significantly higher among females versus males. Also in the univariate logistic regression, male gender is a significant predictor of HCV persistence, and this is supported by previous data [21], [22], [49]. Some previous studies have reported that the rate of SC is higher among females. The apparently higher rate of HCV SC in females is postulated to be due to effect of sex hormone [50] or attributed to genetic and immunological differences between males and females. In our study, gender was not found to be a significant correlate of HCV persistence in multivariate logistic regression analysis. The main reason for this finding may be the fact that IDU is more common among males. This finding implies that the inherent attributes of female gender have probably no direct effect on the natural history of CHCV which is discordant with previous studies [20], [22], [25], [49], [51]. With a similar justification, the seemingly protective role of body piercing in HCV persistence is due to the fact that in our study, women added up to 95.4% of subjects who had pierced while the percentage declined to 1.3% among those who had not, which shows that body piercing is associated with gender and thus is a confounder.

Since the Golestan Cohort Study (GCS) is an aged cohort and the median age of our subjects was 54 years, re-infection might have happened several times and seroconversion might have happened at unknown time; therefore, our cohort may represent a selection of subjects who have recovered from multiple bouts of HCV infections. Studies in older HCV infected subjects documented that prior clearance of HCV decreases the rate of persistent re-infection compared to initial infection [29], [45]; therefore, the duration of re-infection in subjects who have cleared multiple infections may be progressively shorter with each re-infection and this may have been the case in our study too but longer follow up is required to document this claim.

Lastly we compared the rate of SC in Turkmens versus non-Turkmens and in Turkmens versus immigrants from the southern province of Sistan and Balouchestan, who are the second major ethnic group residing in Golestan. Contrary to other studies, we did not find significant difference in the rate of SC between different ethnic groups residing in the province of Golestan. [12], [21], [24].

The main strengths of our study are the prospective population-based design and the large sample size which have been tested. The main limitation is the absence of young subjects in study population. Other limitation includes absence of HCV genotype data.

In conclusion this study revealed a 38.5% spontaneous clearance rate among HCV infected subjects in Golestan. We were able to demonstrate that illicit drug use, either Injecting or non-Injecting, are the main correlates of HCV persistence in Golestan province. Harm reduction interventions including distribution of sterile syringes among IDUs, can be an effective strategy to prevent HCV infection and re-infection in this population, which may play an important role in reducing the burden of HCV infection in Iran. Further studies which include younger age populations are necessary in future.

## Materials and Methods

The study was approved by the ethics committee of Digestive Disease Research Center, Tehran University of Medical Sciences. Written informed consent was obtained from all participants. To determine the rate of HCV SC, we used serum samples stored in biorepository of GCS. The details of GCS have been reported elsewhere [52]. In short, GCS was originally designed to investigate the burden and etiology of upper gastro-intestinal cancers in Northeast of Iran. A total of 50,045 subjects aged 40–75 have been recruited from 3 main districts of the Golestan province in southeast of the Caspian Sea and have been followed-up for 7 years. Subjects have been selected by multi-stage systematic cluster random sampling. Tissue samples including blood, hair, and nail have been collected and stored. The demographic characteristics of the GCS subjects are demonstrated in table 3.

**Table 3:** The demographic characteristics of GCS subjects (N = 49,338) whose serum were available for HCV testing.

Variable		Number (%)
Gender	Female	28,397 (57.6)
	Male	20,941 (42.4)
Age	40–45 years	14,061 (28.5)
	46–55 years	19,735 (40.0)
	>55 years	15,542 (31.5)
Place of Residence	Urban	9,868 (20.0)
	Rural	39,470 (80.0)
Ethinicty	Turkmen	36,707 (74.4)
	Non-Turkmen	12,631 (25.6)

Abbreviations: GCS: Golestan Cohort Study; HCV: Hepatitis C Virus.

For the purpose of the current study, stored serum samples of 49,338 subjects were available for HCV study by ELISA (Diapro, Italy). Positive subjects were contacted via telephone and invited to refer for fresh blood sample collection. Invited subjects who did not respond or were not able to come but did consent to participate in study were visited at home and their blood sample was taken. Samples were transferred in +4°C temperature which took less than 4 hours from collection site to −80°C freezers. No subject was missed.

The newly collected fresh blood samples were tested by ELISA again. All ELISA positive subjects were then tested by Recombinant Immunoblot Assay (RIBA) (MT Company, Germany). RIBA negative subjects were labeled as False Positives. RIBA positive subjects were further tested by Ribonucleic Acid Polymerase Chain Reaction (PCR) (STRPTM Hepatitis C Virus Detection Kit from Cinna Gen- Iran). To test blood samples by PCR, an unthawed sample was used for QubasTaqman with detection limit of 50 copy number.

**Figure 1:**
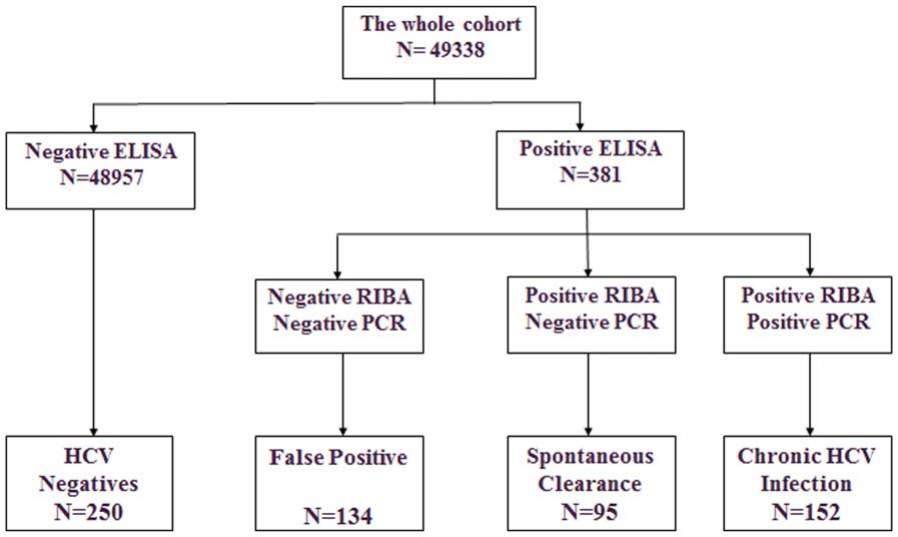
Classification of subjects into cases of chronic HCV infection, Spontaneous Clearance, false positives and HCV negatives.

Six months later, we again performed both RIBA and PCR on subjects who had tested positive for RIBA in the first session. RIBA and PCR positive subjects were classified as cases of chronic HCV infection and RIBA positive but PCR negative subjects were labeled as cases of SC. Control subjects were randomly selected from the bio-repository of the whole cohort (Figure 1).

Demographic data for all subjects were extracted from the GCS database. Data on history of exposure were similarly extracted from the database for HCV negatives. All HCV positive subjects were questioned again about risk factors at the time blood sample was collected.

All analyses were carried out using the statistical software package SPSS for Windows version 18 (Chicago: SPSS Inc., USA). Continuous variables are summarized as mean ± standard deviation of the mean (SD) and categorical variables as frequency and percentage unless otherwise stated. Logistic regression models were used to investigate correlates of HCV acquisition and HCV persistence. The selected multivariate model was backward Wald in which, variables that were significant in univariate model were included. P-values less than 0.05 were considered statistically significant.
